# The interaction of lubricin/proteoglycan 4 (PRG4) with toll-like receptors 2 and 4: an anti-inflammatory role of PRG4 in synovial fluid

**DOI:** 10.1186/s13075-015-0877-x

**Published:** 2015-12-04

**Authors:** Ali Alquraini, Steven Garguilo, Gerard D’Souza, Ling X. Zhang, Tannin A. Schmidt, Gregory D. Jay, Khaled A. Elsaid

**Affiliations:** Department of Pharmaceutical Sciences, School of Pharmacy, MCPHS University, 179 Longwood Ave, Boston, MA 02115 USA; Department of Emergency Medicine, Rhode Island Hospital, Providence, RI USA; Faculty of Kinesiology and Schulich School of Engineering, University of Calgary, Calgary, Canada; Department of Biomedical Engineering, Brown University, Providence, RI USA

**Keywords:** Lubricin, Proteoglycan-4, Toll-like receptors 2 and 4, Arthritis

## Abstract

**Background:**

Lubricin/proteoglycan-4 (PRG4) is a mucinous glycoprotein secreted by synovial fibroblasts and superficial zone chondrocytes. PRG4 has a homeostatic multifaceted role in the joint. PRG4 intra-articular treatment retards progression of cartilage degeneration in pre-clinical posttraumatic osteoarthritis models. The objective of this study is to evaluate the binding of recombinant human PRG4 (rhPRG4) and native human PRG4 (nhPRG4) to toll-like receptors 2 and 4 (TLR2 and TLR4) and whether this interaction underpins a PRG4 anti-inflammatory role in synovial fluid (SF) from patients with osteoarthritis (OA) and rheumatoid arthritis (RA).

**Methods:**

rhPRG4 and nhPRG4 binding to TLR2 and TLR4 was evaluated using a direct enzyme linked immunosorbent assay (ELISA). Association of rhPRG4 with TLR2 and TLR4 overexpressing human embryonic kidney (HEK) cells was studied by flow cytometry. Activation of TLR2 and TLR4 on HEK cells by agonists Pam3CSK4 and lipopolysaccharide (LPS) was studied in the absence or presence of nhPRG4 at 50, 100 and 150 μg/ml. Activation of TLR2 and TLR4 by OA SF and RA SF and the effect of nhPRG4 SF treatment on receptor activation was assessed. PRG4 was immunoprecipitated from pooled OA and RA SF. TLR2 and TLR4 activation by pooled OA and RA SF with or without PRG4 immunoprecipitation was compared.

**Results:**

rhPRG4 and nhPRG4 exhibited concentration-dependent binding to TLR2 and TLR4. rhPRG4 associated with TLR2- and TLR4-HEK cells in a time-dependent manner. Co-incubation of nhPRG4 (50, 100 and 150 μg/ml) and Pam3CSK4 or LPS reduced TLR2 or TLR4 activation compared to Pam3CSK4 or LPS alone (*p* <0.05). OA SF and RA SF activated TLR2 and TLR4 and nhPRG4 treatment reduced SF-induced receptor activation (*p* <0.001). PRG4 depletion by immunoprecipitation significantly increased TLR2 activation by OA SF and RA SF (*p* <0.001).

**Conclusion:**

PRG4 binds to TLR2 and TLR4 and this binding mediates a novel anti-inflammatory role for PRG4.

## Background

Lubricin/proteoglycan-4 (PRG4) is a glycoprotein secreted from synovial fibroblasts and superficial zone chondrocytes that has a multifaceted function including boundary lubrication, resulting in lowering of friction between apposed cartilage surfaces [1–9]. PRG4 is abundant in the synovial fluid (SF) and its levels are reduced in SF from patients with inflammatory arthropathies [10, 11]. The homeostatic role of PRG4 in the joint is evidenced by the ability of purified native protein, recombinant full-length or truncated PRG4 to retard cartilage degeneration, enhance cartilage repair and reduce chondrocyte apoptosis in preclinical surgically induced osteoarthritis (OA) models [12–17].

Recently, we have demonstrated that recombinant human PRG4 (rhPRG4) binds to recombinant human CD44 receptor in a concentration-dependent manner [18]. The interaction of rhPRG4 and CD44 on the surface of rheumatoid arthritis fibroblast-like synoviocytes (RA-FLS) results in inhibition of nuclear factor kappa B (NFκB) nuclear translocation and inhibition of proinflammatory-cytokine-induced RA-FLS proliferation [18]. PRG4 from RA SF binds L-selectin and coats the surface of polymorphonuclear cells (PMNs) [19, 20].

Given the emerging evidence of a biological role for PRG4, we hypothesized that PRG4 acts as an endogenous anti-inflammatory agent in SF through its ability to interact with multiple receptor families. The objective of this investigation was to evaluate the ability of rhPRG4 and native human PRG4 (nhPRG4) to bind to toll-like receptors 2 and 4 (TLR2 and TLR4) and whether this interaction will provide an anti-inflammatory effect by preventing TLR2 and TLR4 activation by agonists and by ligands in SF from patients with OA and RA.

## Methods

### Binding of recombinant and native human PRG4 to TLR2 and TLR4 using a direct enzyme-linked immunosorbent assay (ELISA) approach

High-binding microtiter plates (Corning, Sigma Aldrich, St. Louis, MO, USA) were used to coat recombinant human TLR2 or TLR4 overnight at 4 °C (R&D Systems, Minneapolis, MN, USA). Each well received 100 μL of TLR2 or TLR4 (1 μg/mL each) in phosphate-buffered saline (PBS). Wells that were coated with TLR2 or TLR4 or corresponding uncoated control wells were blocked with 2 % bovine serum albumin (BSA; 300 μL per well) for 2 h at room temperature. rhPRG4 or nhPRG4 were added at 50, 10, 1, and 0.1 μg/mL (100 μL per well) and incubated for 1 h at room temperature. rhPRG4 is a full-length product produced by CHO-M cells (Lubris, Framingham, MA, USA) [21]. nhPRG4 is purified from culture supernatants of human FLS as previously described [13]. rhPRG4 and nhPRG4 have similar immunoreactivity towards anti-PRG4 antibodies and have similar molecular weights assessed by gel electrophoresis [21]. Following washing with PBS + 0.05 % tween 20, anti-PRG4 antibody (9G3, EMD Millipore, Billerica, MA, USA) [22] was added at 1:1,000 dilution in PBS + 0.05 % tween 20 with each well receiving 100 μL of the antibody solution and incubated for 1 h at room temperature. Wells were washed with PBS + 0.05 % tween 20 and sheep anti-mouse IgG-horseradish peroxidase (HRP) was added (1:5,000 dilution; 100 μL per well) and incubated at room temperature for 1 h. Following washing with PBS + 0.05 % tween 20, the assay was developed using 1-step Turbo TMB ELISA reagent (ThermoFisher Scientific, Waltham, MA, USA) and the absorbance was measured at 450 nm. Data are presented as fold-change in the 450 nm absorbance values above uncoated wells (non-specific binding). Data represent the mean ± SD of four independent experiments with at least triplicate wells per group.

### Association of fluorescently labeled rhPRG4 with TLR2- and TLR4-expressing human embryonic kidney (HEK) cells using flow cytometry

rhPRG4 was labeled with Rhodamine using a commercially available labeling kit according to the manufacturer’s recommendation (Pierce NHS–Rhodamine Antibody Labeling Kit, ThermoFisher Scientific). Association of rhPRG4 with TLR2 and TLR4 was studied following incubation of Rhodamine-rhPRG4 with HEK-Blue hTLR2 and HEK-Blue hTLR4 cells (Invivogen, San Diego, CA, USA). TLR2-HEK and TLR4-HEK cells are genetically engineered reporter cells derived from HEK cells by co-transfection of the human TLR2 with an inducible secreted embryonic alkaline phosphatase (SEAP) or the co-transfection of the human TLR4, MD-2 and CD14 co-receptor genes with the SEAP reporter gene. Activation of TLR2 or TLR4 results in nuclear translocation of NFκB and expression of SEAP, whose activity can be detected in the culture media.

A total of 100,000 TLR2-HEK or TLR4-HEK cells were plated per well in sterile tissue culture plates. Cells were treated with Rhodamine-rhPRG4 (20 μg/mL) for 12 or 24 h. Following incubation, cells were harvested using cell scrapers, pelleted and washed with PBS. Cell associated fluorescence was analyzed using Guava easyCyte Flow Cytometer (EMD Millipore). Cell-associated fluorescence was measured across independent experiments using the same acquisition parameters. A threshold was set at red fluorescence intensity = 10, and cells that displayed fluorescence intensity higher than the threshold were counted as positively associating with Rhodamine rhPRG4. Data are presented as percentage positive cells across different treatments. Data represent the mean ± SD of four independent experiments.

### Inhibition of TLR2 and TLR4 activation by nhPRG4

In sterile 96-well plates (Corning, Sigma Aldrich), 25,000 cells of either TLR2-HEK or TLR4- HEK cells were plated per well. Cells were suspended in HEK Blue detection media (Invivogen) and the total volume in each well was 200 μL. HEK Blue detection media contains a SEAP substrate that allows the colorimetric detection of SEAP activity. TLR2-HEK cells were treated with a TLR2 agonist, Pam3CSK4 (Invivogen) (50 ng/mL) in the absence or presence of nhPRG4 (50, 100, and 150 μg/mL) and cells were incubated at 37 °C for 24 h. Pam3CSK4 was diluted in endotoxin-free water and subsequently added to the cells in HEK detection media. The 630 nm absorbance was measured and normalized to untreated control cells. Data are presented as fold-change in 630 nm above untreated control cells. Data represent the mean ± SD of four independent experiments with at least duplicate wells per experimental group. TLR4-HEK cells were treated with 50 ng/mL lipopolysaccharide (LPS; Invivogen) in the absence or presence of nhPRG4 (50, 100 and 150 μg/mL) and cells were incubated at 37 °C for 20 h. LPS was diluted in endotoxin-free water and subsequently added to the cells in HEK detection media. The 630 nm absorbance was measured and normalized to untreated control cells. Data are presented as fold-change in 630 nm above untreated control cells. Data represent the mean ± SD of four independent experiments with at least duplicate wells per experimental group.

### Activation of TLR2 and TLR4 by OA and RA SF and the effect of nhPRG4 treatment

Samples from RA (n = 10), OA (n = 8) and normal (n = 3) SF specimens were used throughout this study. SF aliquots from five RA patients were kindly provided by Dr. Stefan Lohmander. These samples were collected with patients’ consent and approved by the central ethical review board at Lund University, Lund, Sweden. The remainder of the SF specimens were acquired from Articular Engineering, Northbrook, IL, USA, following knee replacement surgery or from donors within 24 h of death, collected with partner site Institutional Review Board (IRB) approval with informed written consent from the donor or nearest relative. This study was approved by the IRB at the Massachusetts College of Pharmacy and Health Sciences. Nine of the RA patients were female. The median age (interquartile range) of the group was 73 (57 to 75) years. Eight patients were on a methotrexate regimen, among whom four patients were on combined treatment with a disease-modifying biologic agent. Five of the OA patients were female. The median age (interquartile range) of the group was 65 (59 to 69) years. Normal SF specimens were obtained from subjects with no clinical history of joint disease or arthritis. Two normal subjects were male (56 and 62 years old) and one was female (35 years old). OA, RA and normal SF specimens (7.5 μL per well; 3.75 % SF dilution) were incubated with TLR2-HEK cells (25,000 cells per well in HEK Blue detection media). The final volume in each well was 200 μL. Cells were incubated with the SF samples for 48 h at 37 °C. The 630 nm absorbance was measured and normalized to the 630 nm absorbance values of untreated control cells. A similar approach was adopted for measuring the activation of TLR4-HEK cells with OA, RA and normal SF.

In a separate set of experiments, OA SF samples (n = 8) or RA SF samples (n = 5) (7.5 μL per well; 3.75 % SF dilution) were incubated with TLR2-HEK cells (25,000 cells per well in HEK Blue detection media) in the absence or presence of nhPRG4 (100 μg/mL). The final volume in each well was 200 μL. Cells were incubated with the SF samples for 48 h at 37 °C. The 630 nm absorbance was measured and normalized to the 630 nm absorbance values of untreated control cells. Similarly, OA SF samples (n = 8) or RA SF samples (n = 5) (7.5 μL per well; 3.75 % SF dilution) were incubated with TLR4-HEK cells (25,000 cells per well in HEK Blue detection media) in the absence or presence of nhPRG4 (150 μg/mL). The final volume in each well was 200 μL. Cells were incubated with the SF samples for 48 h at 37 °C. The 630 nm absorbance was measured and normalized to 630 nm absorbance values of untreated control cells.

### Immunoprecipitation of PRG4 from OA and RA SF

SF aliquots from OA patients (n = 5) and RA patients (n = 5) were pooled and digested with *Streptomyces* hylauronidase (Sigma Aldrich) at a final enzyme concentration of 3 units/ml for 3 h at 37 °C. Digested pooled OA and pooled RA SF underwent two rounds of PRG4 depletion by immunoprecipitation. In each round, SF aliquots were incubated with monoclonal anti-PRG4 antibody, 9G3 (1:100 dilution) for 2 h at 37 °C. Subsequently, G-protein-coupled Dynabeads (ThermoFisher Scientific) were added to the OA and RA SF (1.5 mg) and incubated at 37 °C for 1 h followed by magnetic separation (DynaMag, ThermoFisher Scientific). Fresh magnetic beads were added (1.5 mg) and magnetic separation was conducted as described above. To confirm PRG4 depletion from OA and RA SF, SF aliquots were assayed for PRG4 content using a PRG4 inhibition ELISA as previously described [23].

### Effect of PRG4 removal on TLR2 and TLR4 activation by OA and RA SF

PRG4-immunoprecipitated pooled OA or RA SF and pooled OA or RA SF, at 1.0, 7.5, 10.0 and 20.0 μL, were incubated with TLR2-HEK cells (25,000 cells per well in HEK Blue detection media) for 24 h at 37 °C. The final volume in each well was 200 μL, corresponding to SF dilution of 0.5, 3.75, 5.0 and 10.0 %. The 630 nm absorbance was measured and normalized to untreated control cells. Similarly, PRG4-immunoprecipitated pooled OA or RA SF and pooled OA or RA SF were incubated with TLR4-HEK cells as described above.

In a separate set of experiments, PRG4-immunoprecipitated pooled OA or RA SF and pooled OA or RA SF at 5 % SF dilution were incubated with TLR2-HEK cells (25,000 cells per well in HEK Blue detection media) in the absence or presence of nhPRG4 (100, 200 and 300 μg/mL) for 24 h at 37 °C, followed by absorbance measurement as described above.

### Statistical analyses

Variables were initially tested for normality and equal variances. Variables that satisfied both assumptions were tested for statistical significance using Student’s *t* test or analysis of variance (ANOVA) with Tukey’s post-hoc test for two-group and more than two-group comparisons, respectively. Variables that did not satisfy the normality assumption were tested using the Mann–Whitney *U* test or ANOVA on the ranks. The level of statistical significance was set at α = 0.05. Data are presented as the mean ± SD. Unless otherwise specified, data represent the mean of three independent experiments with duplicate wells per experimental group.

## Results

### Binding of rhPRG4 and nhPRG4 to immobilized TLR2 and TLR4

**Fig. 1:**
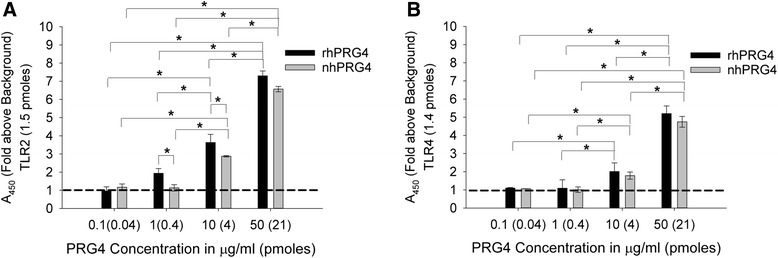
Binding of recombinant human proteoglycan-4 (*rhPRG4*) and native human PRG4 (*nhPRG4*) to recombinant toll-like receptors 2 and 4 (*TLR2* and TLR4) using a direct binding enzyme linked immunosorbent assay. PRG4 concentrations are reported in μg/mL and pmoles per well, based on a predicted molecular weight of 240 kDa. TLR2 and TLR4 are expressed as pmoles coated per well, based on predicted molecular weights of 66 and 70 kDa, respectively. The 450 nm absorbance values were normalized to non-specific binding. Data represent the mean ± SD from four independent experiments. *Dashed line* represents non-specific binding (background). **a** Concentration-dependent binding of rhPRG4 and nhPRG4 to immobilized TLR2. rhPRG4 exhibited a concentration-dependent binding at 50, 10 and 1 μg/mL. nhPRG4 exhibited a concentration-dependent binding at 50 and 10 μg/mL; *p <0.001. **b** Concentration-dependent binding of rhPRG4 and nhPRG4 to immobilized TLR4. rhPRG4 and nhPRG4 exhibited concentration-dependent binding at 50 and 10 μg/mL; *p <0.001

The concentration-dependent binding of rhPRG4 and nhPRG4 to TLR2 is shown in Fig. 1a. rhPRG4 and nhPRG4 concentrations are reported in μg/Ml and PRG4 in pmoles, based on a predicted molecular weight of 240 kDa [18]. rhPRG4 (1, 10, and 50 μg/mL), corresponding to 0.4, 4 and 21 pmoles, significantly binds to TLR2-coated wells (1.5 pmoles per well based on a predicted molecular weight of 66 kDa) compared to uncoated wells (*p* <0.001). nhPRG4 (10 and 50 μg/mL), corresponding to 4 and 21 pmoles, significantly binds to TLR2-coated wells compared to uncoated wells (*p* <0.001). There was concentration-dependent binding of rhPRG4 to TLR2, with significant reduction in binding to TLR2 at 1 μg/mL compared to the 10 and 50 μg/mL (*p* <0.001). Similarly, rhPRG4 (10 μg/mL) displayed significant reduction in binding to TLR2 compared to rhPRG4 (50 μg/mL). nhPRG4 displayed a reduction in TLR2 binding at 10 μg/mL compared to 50 μg/mL. rhPRG4 demonstrated significantly higher binding to TLR2 compared to nhPRG4 at 1 and 10 μg/mL (*p* <0.001).

rhPRG4 (10 and 50 μg/mL), corresponding to 4 and 21 pmoles, significantly binds to TLR4-oated wells (1.4 pmoles per well based on a predicted molecular weight of 70 kDa) compared to uncoated wells (*p* <0.001) (Fig. 1b). nhPRG4 (10 and 50 μg/mL), corresponding to 4 and 21 pmoles, significantly binds to TLR4 coated wells compared to uncoated wells (*p* <0.001). Both rhPRG4 and nhPRG4 displayed concentration-dependent binding to TLR4 as the 50 μg/mL in both forms of the protein significantly binds to TLR4 compared to 10 μg/mL (*p* <0.001).

### Association of rhPRG4 with TLR2-HEK and TLR4-HEK cells

**Fig. 2:**
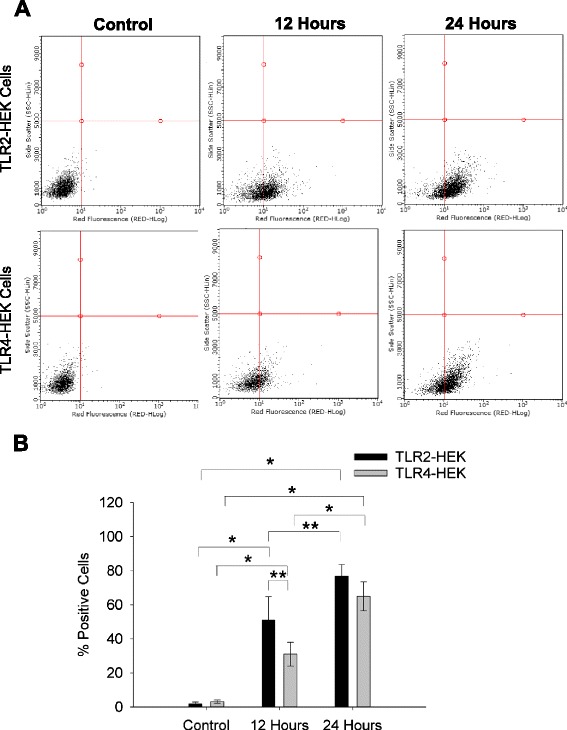
Association of recombinant human proteoglycan-4 (rhPRG4) with toll-like receptor-2 and 4 (TLR2 and TLR4) expressing human embryonic kidney (*TLR2-HEK* and *TLR4-HEK*) cells. Data represent the mean ± SD from four independent experiments. **a** Representative flow cytometry scatter plots of rhodamine-labeled rhPRG4 (20 μg/mL) association with TLR2-HEK and TLR4-HEK cells following incubation for 12 and 24 h. A threshold was set at red fluorescence intensity = 10. Cell-associated fluorescence higher than 10 was considered positive. Following incubation of rhodamine-rhPRG4 with TLR2-HEK and TLR4-HEK cells for 12 or 24 h, cell association was observed. **b** Quantitative analysis of the percentage of positive TLR2-HEK and TLR4-HEK cells following incubation with rhodamine-rhPRG4 (20 μg/mL) for 12 and 24 h. rhPRG4 was significantly associated with TLR2-HEK and TLR4-HEK at 24 h compared to 12 h and controls. The 12-h association between rhPRG4 and TLR2-HEK cells was significantly higher than with TLR4-HEK cells and both were higher than control; *p <0.001, ** *p* <0.05

Representative flow cytometry scatter plots showing time-dependent association of fluorescently labeled rhPRG4 with TLR2-HEK and TLR4-HEK cells are shown in Fig. 2a. Control untreated cell population is located in the lower left quadrant of the graph. Treatment of TLR2-HEK cells with rhPRG4 for 12 and 24 h resulted in a cell population shift to the lower right quadrant indicating that rhPRG4 was associated with TLR2-HEK cells. Similarly, treatment of TLR4-HEK cells with rhPRG4 for 12 and 24 h exhibited a similar shift indicating that rhPRG4 was associated with TLR4-HEK cells.

Quantitative analysis of rhPRG4 association with TLR2-HEK and TLR4-HEK cells is shown in Fig. 2b. At 12 and 24 h, the percentage of TLR2-HEK and TLR4-HEK cells that were associated with rhPRG4 was significantly higher than control (*p* <0.001). For TLR2-HEK, the percentage of cells that associated with rhPRG4 following incubation for 24 h was significantly higher than the corresponding percentage following incubation for 12 h (*p* = 0.011). Similarly, percentage of TLR4-HEK cells that associated with rhPRG4 following incubation for 24 h was significantly higher than the corresponding percentage following incubation for 12 h (*p* <0.001). Following incubation for 12 h, rhPRG4 showed significantly higher association with TLR2-HEK compared to TLR4-HEK cells (*p* <0.001). In contrast, there was no significant difference in rhPRG4 association with TLR2-HEK and TLR4-HEK cells following 24-h treatment (*p* = 0.353).

### nhPRG4 blocks agonist-induced activation of TLR2 and TLR4

**Fig. 3:**
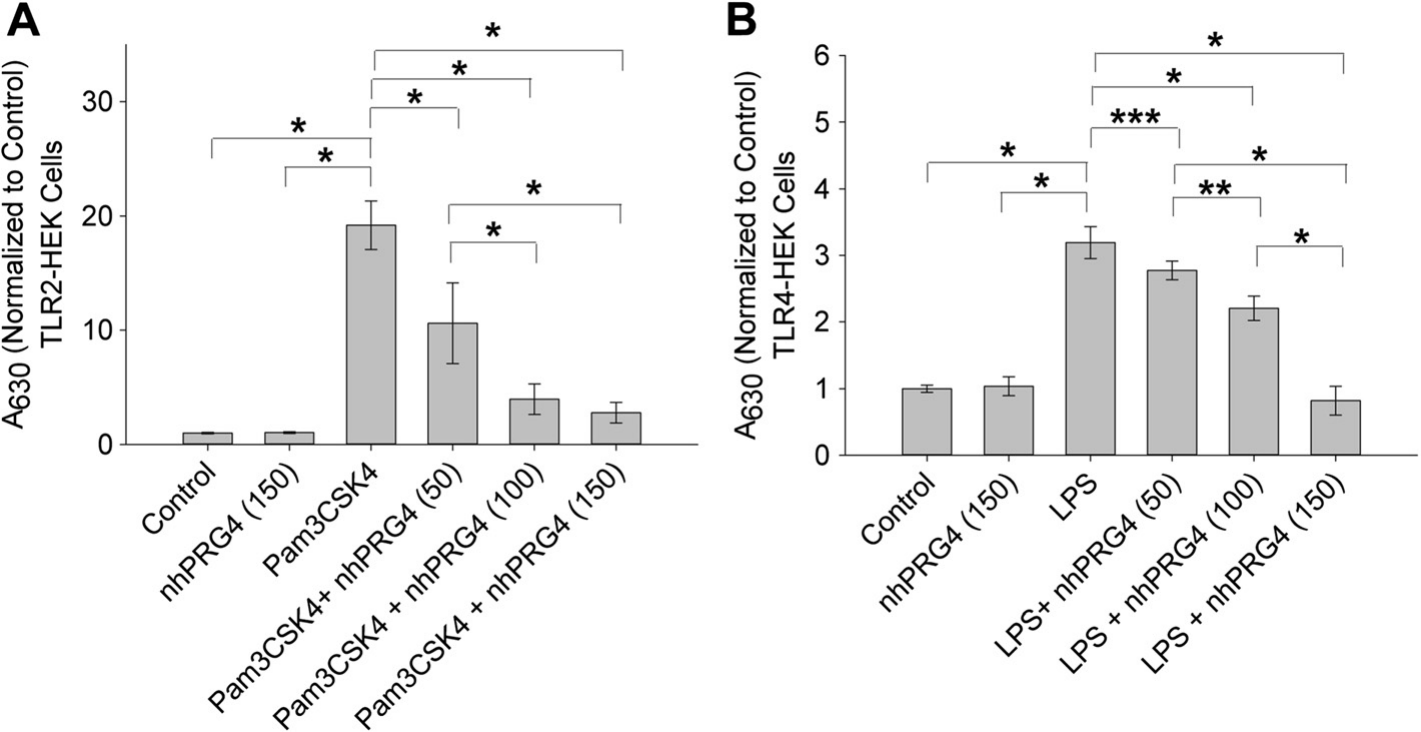
Concentration-dependent effect of native human proteoglycan 4 (*nhPRG*4) on agonist-induced activation of toll-like receptors 2 and 4 (*TLR2* and *TLR4*) expressing human embryonic kidney (*TLR2-HEK* and *TLR4-HEK*) cells. Absorbance values across groups were normalized to untreated cells (control). Data represents the mean ± SD from four independent experiments. **a** Inhibition of Pam3CSK4-induced activation of TLR2 by nhPRG4 treatment. Pam3CSK4 significantly activated TLR2. nhPRG4 (50, 100 and 150 μg/mL) co-incubation significantly reduced Pam3CSK4-induced TLR2 activation. nhPRG4 (100 and 150 μg/mL) treatments were more efficacious in reducing TLR2 activation than nhPRG4 (50 μg/mL). nhPRG4 alone did not stimulate TLR2; *p <0.001. **b** Inhibition of lipopolysaccharide (*LPS*)-induced activation of TLR4 by nhPRG4 treatment. LPS significantly activated TLR4. nhPRG4 (50, 100 and 150 μg/mL) co-incubation significantly reduced LPS-induced TLR4 activation. nhPRG4 (100 and 150 μg/mL) treatments were more efficacious in reducing TLR4 activation than nhPRG4 (50 μg/mL). nhPRG4 (150 μg/mL) treatment was more efficacious in reducing TLR4 activation than nhPRG4 (100 μg/mL). nhPRG4 alone did not stimulate TLR4; *p <0.001, **p <0.01, ***p <0.05

The effect of nhPRG4 treatment on agonist-induced TLR2 activation is shown in Fig. 3a. Pam3CSK4 treatment resulted in significant TLR2 activation compared to control (*p* <0.001). Co-incubation of nhPRG4 (50, 100 and 150 μg/mL) with Pam3CSK4 significantly reduced TLR2 activation compared to Pam3CSK4 only treatment (*p* <0.001). nhPRG4 (100 and 150 μg/mL) treatment was more efficacious in reducing TLR2 activation than nhPRG4 (50 μg/mL) (*p* <0.001, *p* <0.001). nhPRG4 (150 μg/mL) and nhPRG4 (100 μg/mL) treatments were equally efficacious in reducing TLR2 activation (*p* = 0.718). nhPRG4 (150 μg/mL) alone did not stimulate TLR2 receptor compared to untreated control cells.

The effect of nhPRG4 treatment on agonist-induced TLR4 activation is shown in Fig. 3b. LPS treatment resulted in significant TLR4 activation compared to untreated control cells (*p* <0.001). Co-incubation of nhPRG4 (50, 100 and 150 μg/mL) with LPS resulted in a significant reduction in TLR4 activation compared to LPS only treatment (*p* = 0.033, *p* <0.001, *p* <0.001). nhPRG4 (100 and 150 μg/mL) treatment was more efficacious in reducing TLR4 activation than nhPRG4 (50 μg/mL) (*p* = 0.003, *p* <0.001). Similarly, nhPRG4 (150 μg/mL) treatment was more efficacious in reducing TLR4 activation than nhPRG4 (100 μg/mL) (*p* <0.001). nhPRG4 (150 μg/mL) alone did not stimulate TLR4 receptor compared to untreated control cells.

### OA and RA SF activate TLR2 and TLR4 and nhPRG4 treatment blocks SF-mediated activation

**Fig. 4:**
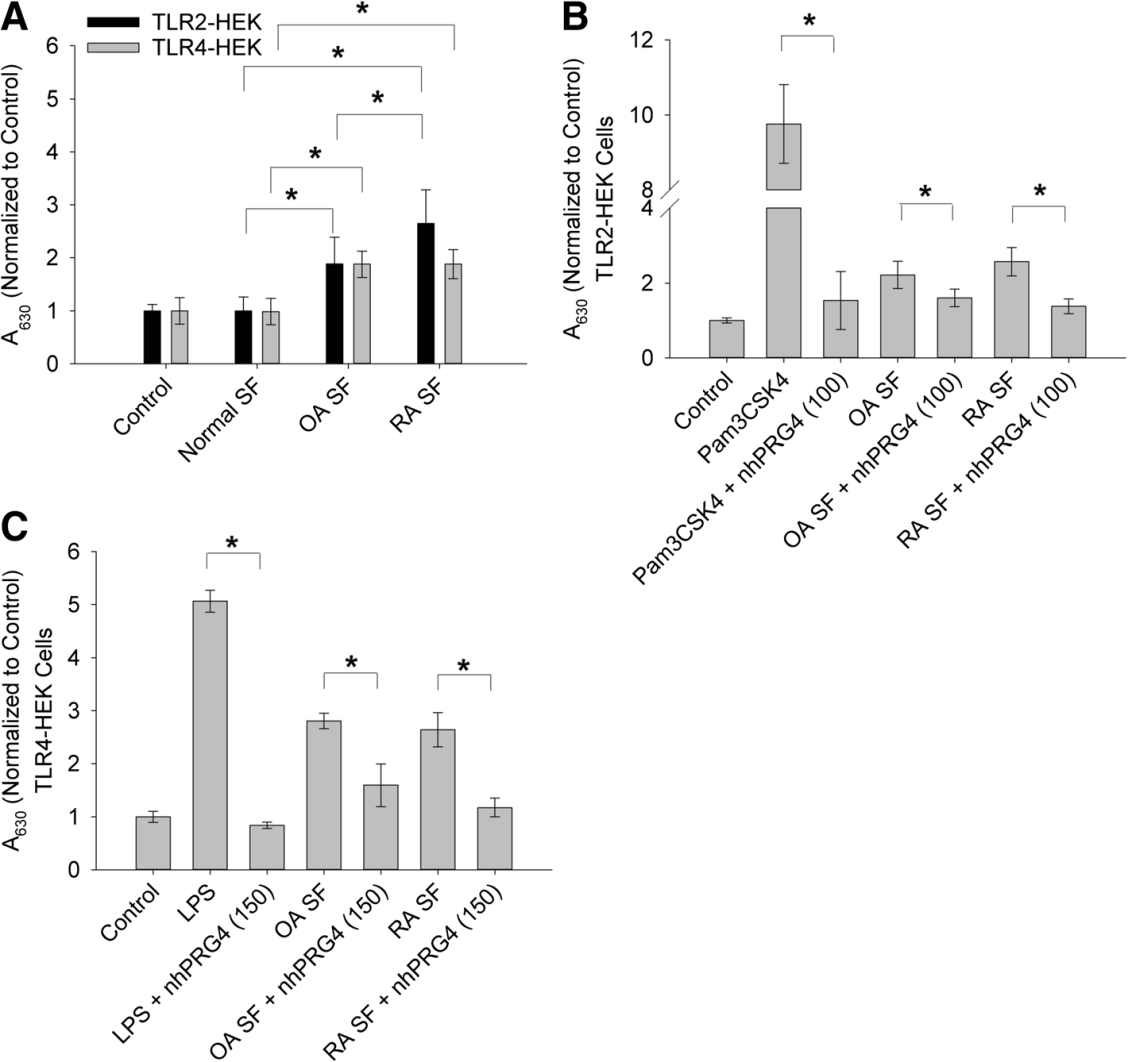
Activation of toll-like receptors 2 and 4 (TLR2 and TLR4) expressing human embryonic kidney (*TLR2-HEK* and *TLR4-HEK*) cells by synovial fluids (*SF*) from subjects with no joint arthropathy (normal; n = 3), patients with osteoarthritis (*OA*; n = 8) and patients with rheumatoid arthritis (*RA*; n = 5) and effect of native human proteoglycan-4 (nhPRG4) treatment. SF samples were incubated with TLR2- or TLR4-HEK cells (3.75 % dilution) at 37 °C for 48 h. Absorbance values across groups were normalized to untreated cells (control). Data represent the mean SD from three independent experiments. **a** Activation of TLR2 and TLR4 by normal, OA and RA SF. Normal SF did not significantly activate TLR2 or TLR4 compared to control. OA SF and RA SF significantly activated TLR2 and TLR4 compared to normal SF and control. RA SF significantly activated TLR2 compared to OA SF; *p <0.001. **b** Impact of nhPRG4 treatment (100 μg/mL) on Pam3CSK4, OA SF and RA SF-induced activation of TLR2. nhPRG4 treatment inhibited Pam3CSK4, OA SF and RA SF-induced TLR2 activation; *p <0.001. **c** Impact of nhPRG4 treatment (150 μg/mL) on lipopolysaccharide (*LPS*), OA SF and RA SF-induced activation of TLR4. nhPRG4 treatment inhibited LPS, OA SF and RA SF-induced TLR4 activation; *p <0.001

Activation of TLR2 and TLR4 by OA SF and RA SF samples is shown in Fig. 4a. Normal SF did not result in TLR2 or TLR4 activation compared to control. RA SF treatment resulted in significantly higher TLR2 activation compared to OA SF and normal SF (*p* <0.001). Similarly, OA SF treatment resulted in a significantly higher TLR2 activation compared to normal SF (*p* <0.001). Both OA and RA SF significantly activated TLR4 compared to normal SF and control (*p* <0.001). There was no significant difference in TLR4 activation between OA SF and RA SF (*p* = 0.786). nhPRG4 (100 μg/mL) treatment significantly reduced TLR2 activation by Pam3CSK4, OA SF and RA SF (*p* <0.001) (Fig. 4b). Similarly, rhPRG4 (150 μg/mL) treatment significantly reduced TLR4 activation by LPS, OA SF and RA SF (*p* <0.001) (Fig. 4c).

### Effect of PRG4 depletion by immunoprecipitation on OA and RA SF-mediated activation of TLR2 and TLR4

**Fig. 5:**
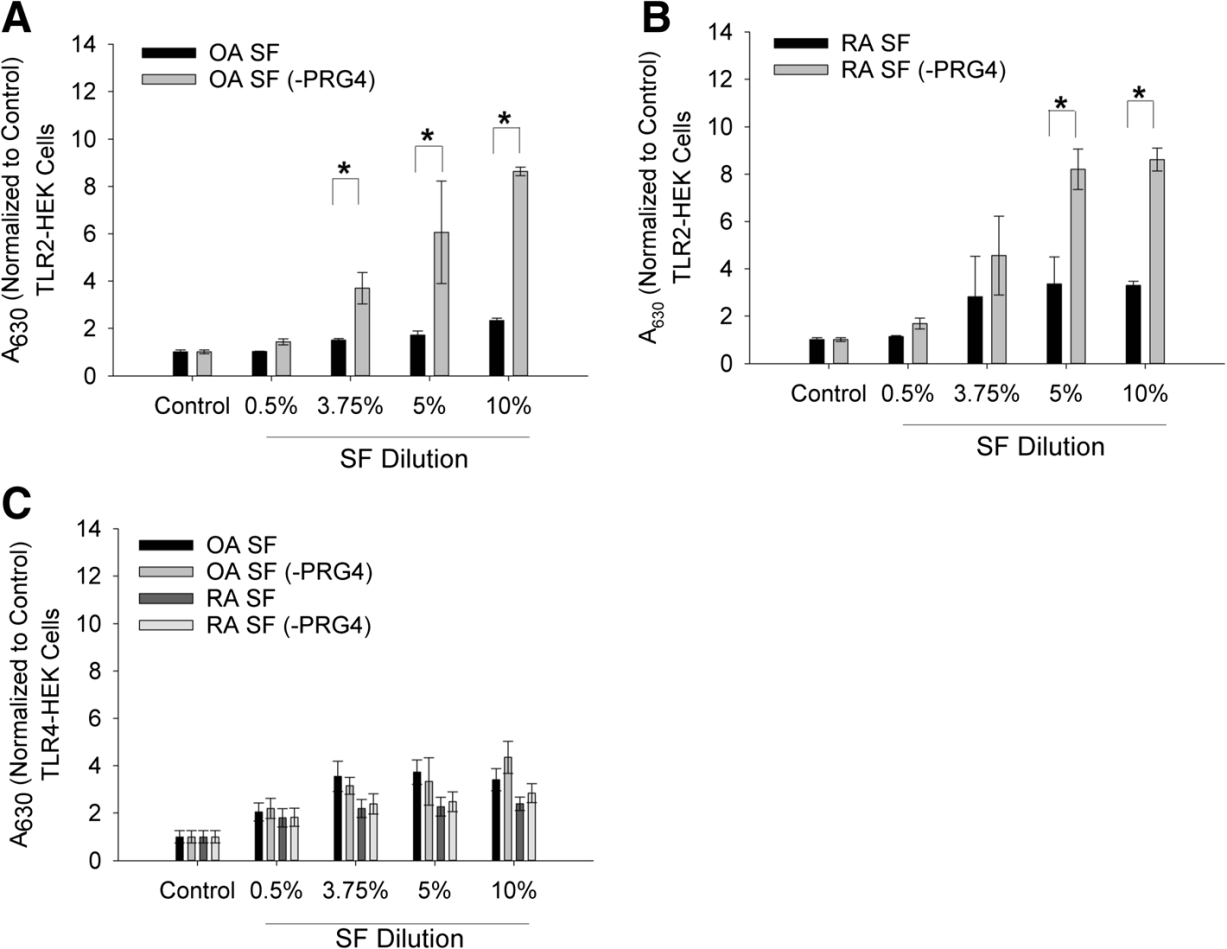
Impact of proteoglycan-4 (*PRG4*) immunoprecipitation on activation of toll-like receptors 2 and 4 (TLR2 and TLR4) expressing human embryonic kidney (*TLR2-HEK* and *TLR4-HEK*) cells by pooled osteoarthritis (OA) (n = 5) and pooled rheumatoid arthritis (RA) (n = 5) synovial fluid (*SF*). *OA* or *RA* SF with or without PRG4 immunoprecipitation were incubated with TLR2-HEK or TLR4-HEK cells (0.5, 3.75, 5.0 and 10.0 % dilution) at 37 °C for 24 h. The 630 nm absorbance values across experimental groups were normalized to untreated controls. Data represent the mean ± SD from three independent experiments. **a** Impact of PRG4 immunoprecipitation on TLR2 activation by OA SF. TLR2 activation was significantly higher in PRG4-immunoprecipitated OA SF (OA SF (-PRG4)) compared to OA SF at 3.75, 5.0 and 10.0 %; *p <0.001. **b** Impact of PRG4 immunoprecipitation on TLR2 activation by RA SF. TLR2 activation was significantly higher in PRG4 immunoprecipitated RA SF (RA SF (-PRG4)) compared to RA SF at 5 and 10 %; *p <0.001. **c** Impact of PRG4 immunoprecipitation on TLR4 activation by OA SF and RA SF. There was no significant difference in TLR4 activation between OA SF and OA SF following PRG4 immunoprecipitation (OA SF (-PRG4)) across any SF dilutions. There was no significant difference in TLR4 activation between RA SF and RA SF following PRG4 immunoprecipitation (RA SF (-PRG4)) across any SF dilutions

PRG4 depletion from pooled OA and RA SF was confirmed using PRG4 ELISA. The mean PRG4 concentration in OA SF was 280.43 ± 14.76 μg/mL compared to 25.11 ± 3.18 μg/mL in PRG4-depleted OA SF (OA SF (-PRG4)). The mean PRG4 concentration in RA SF was 226 ± 4.09 μg/mL compared to 32.17 ± 5.57 μg/mL in PRG4-depleted RA SF (RA SF (-PRG4)). The effect of PRG4 depletion on TLR2 activation by OA SF is shown in Fig. 5a. There was no significant difference in TLR2 activation between OA SF and OA SF (-PRG4) at 0.5 % SF dilution. At 3.75, 5 and 10 % SF dilution, OA SF (-PRG4) treatment resulted in significantly higher TLR2 activation compared to OA SF treatment and untreated controls (*p* <0.001). Similarly, at 3.75, 5 and 10 % SF dilution, OA SF treatment resulted in significantly higher TLR2 activation compared to untreated control cells (*p* <0.001). The effect of PRG4 depletion on TLR2 activation by RA SF is shown in Fig. 5b. There was no significant difference in TLR2 activation between RA SF and RA SF (-PRG4) at 0.5 and 3.75 % SF dilution. At 5 and 10 % SF dilution, RA SF (-PRG4) treatment resulted in increased TLR2 activation compared to RA SF treatment (*p* <0.001). Similarly, at 3.75, 5 and 10 % SF dilution, RA SF treatment resulted in significantly higher TLR2 activation compared to untreated control cells (*p* <0.001).

The effect of PRG4 depletion on TLR4 activation by OA and RA SF is shown in Fig. 5c. At 0.5, 3.75, 5.0 and 10.0 % SF dilution, OA SF and OA SF (-PRG4) treatments resulted in significantly higher TLR4 activation compared to untreated controls (*p* <0.001). There was no significant difference in TLR4 activation between OA SF and OA SF (-PRG4) across the various SF dilutions. RA SF and RA SF (-PRG4) significantly activated TLR4 compared to untreated controls at all the SF dilutions (*p* <0.001). PRG4 depletion from pooled RA SF did not result in a significant change in TLR4 activation compared to non-depleted RA SF across all SF dilutions.

**Fig. 6:**
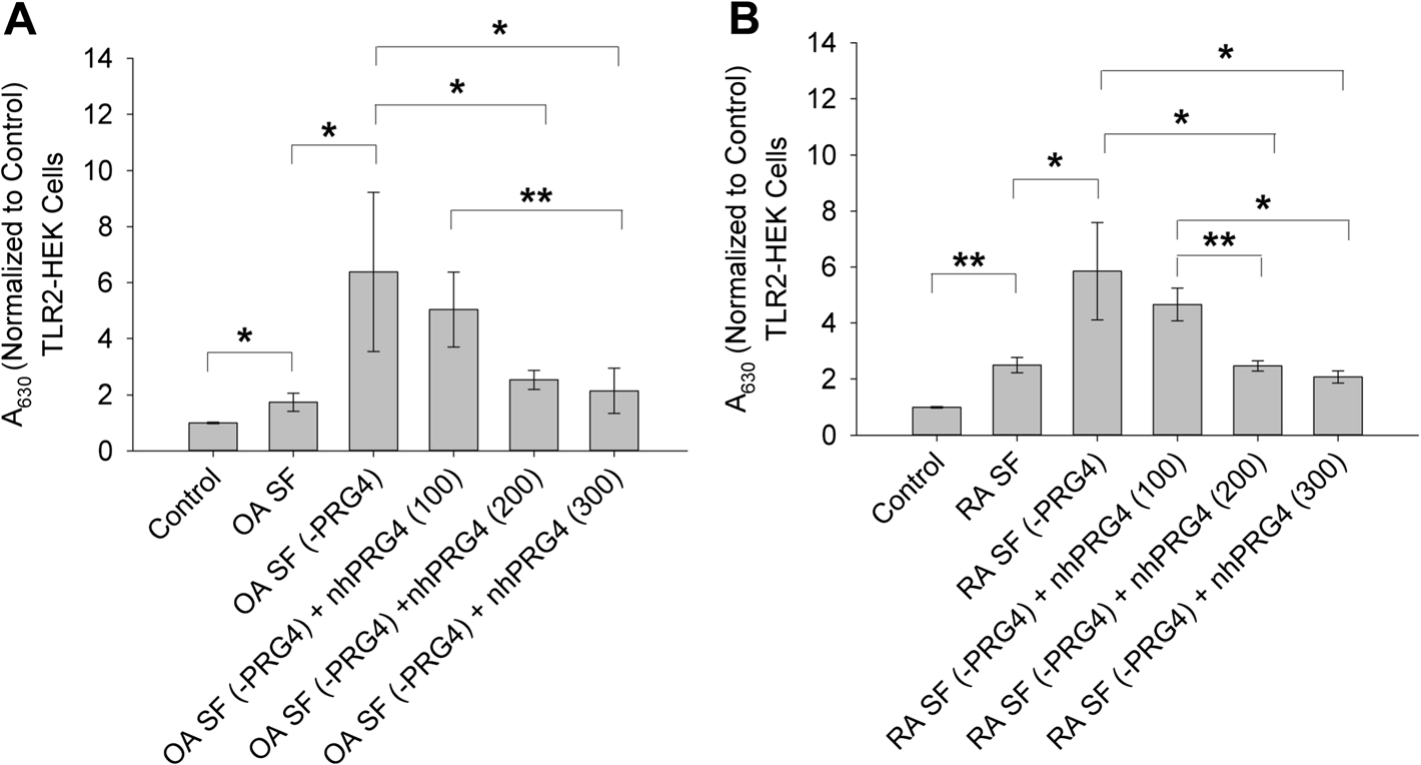
Impact of native human proteoglycan-4 (*nhPRG4*) treatment (100, 200 and 300 μg/mL) on toll-like receptor 2 (*TLR2*) activation by pooled osteoarthritis (*OA*) synovial fluid (*SF*) and pooled rheumatoid arthritis (*RA*) SF following proteoglycan-4 (*PRG4*) immunoprecipitation. TLR2-expressing human embryonic kidney (*TLR2-HEK*) cells were incubated with SF (5 % dilution for 24 h) in the absence or presence of nhPRG4. The 630 nm absorbance values across experimental groups were normalized to untreated controls. Data represent the mean ± SD from three independent experiments. **a** Impact of nhPRG4 on TLR2 activation of PRG4-immunoprecipitated OA SF (OA SF (-PRG4)). nhPRG4 treatments (200, 300 μg/mL) significantly reduced TLR2 activation by OA SF (-PRG4). nhPRG4 (300 μg/mL) treatment was more efficacious in reducing TLR2 activation than nhPRG4 (100 μg/mL); *p <0.001; **p <0.05. **b** Impact of nhPRG4 on TLR2 activation of PRG4-immunoprecipitated RA SF (RA SF (-PRG4)). nhPRG4 treatments (200, 300 μg/mL) significantly reduced TLR2 activation by RA SF (-PRG4). nhPRG4 (200 and 300 μg/mL) treatment was more efficacious in reducing TLR2 activation than nhPRG4 (100 μg/mlL); *p <0.001; **p <0.01

The impact of nhPRG4 supplementation on TLR2 activation by PRG4-depleted OA SF is shown in Fig. 6a. OA SF treatment resulted in significantly higher TLR2 activation compared to untreated controls (*p* <0.001). Likewise, OA SF (-PRG4) treatment resulted in significantly higher TLR2 activation compared to OA SF and untreated controls (*p* <0.001). There was no significant difference between TLR2 activation in the OA SF (-PRG4) and OA SF (-PRG4) + nhPRG4 (100 μg/mL) groups (*p* = 0.454). TLR2 activation was significantly reduced in the OA SF (-PRG4) + nhPRG4 (200 or 300 μg/mL) compared to OA SF (-PRG4) (*p* <0.001). There was no significant difference in TLR2 activation between OA SF (-PRG4) + nhPRG4 (300 μg/mL) and OA SF (-PRG4) + nhPRG4 (200 μg/mL) (*p* = 0.312). There was a trend towards a significant reduction in TLR2 activation of OA SF (-PRG4) + nhPRG4 (200 μg/mL) and OA SF (-PRG4) + nhPRG4 (100 μg/mL) (*p* = 0.051).

The impact of nhPRG4 supplementation on TLR2 activation by PRG4-depleted RA SF is shown in Fig. 6b. RA SF treatment resulted in a significant activation of TLR2 compared to untreated controls (*p* = 0.003). RA SF (-PRG4) treatment significantly activated TLR2 compared to RA SF and untreated controls (*p* <0.001). There was no significant difference in TLR2 activation between RA SF (-PRG4) and RA SF (-PRG4) + nhPRG4 (100 μg/mL) (*p* = 0.155). TLR2 activation was significantly reduced in RA SF (-PRG4) + nhPRG4 (200 or 300 μg/mL) compared to RA SF (-PRG4) (*p* <0.001). Similarly, TLR2 activation in RA SF (-PRG4) + nhPRG4 (200 or 300 μg/mL) was significantly reduced compared to RA SF (-PRG4) + nhPRG4 (100 μg/mL) (*p* = 0.004; *p* <0.001). There was no significant difference in TLR2 activation between RA SF (-PRG4) + nhPRG4 (200 μg/mL) and RA SF (-PRG4) + nhPRG4 (300 μg/mL) (*p* = 0.672).

## Discussion

The TLR family is a large family of receptors, with at least 11 members that have been identified thus far [24]. The TLRs play an important role in the host defense mechanism as part of the innate immune response. The different members of the TLR family have evolved to recognize pathogen-associated molecular patterns (PAMPs), including cell wall components of Gram-positive, Gram-negative bacteria, viruses and fungi [24]. TLRs are also stimulated by damage-associated molecular patterns (DAMPs). DAMPs are host-derived proteins including extracellular matrix components, and macromolecule fragments that are released due to tissue injury and cell death and can activate the TLRs [25]. Examples of DAMPs that were shown to activate TLRs, specifically TLR2 or TLR4, in the joint environment include biglycan, high mobility group box 1 protein (HMGB1), glycoprotein 96 (gp96), fibronectin, low molecular weight hyaluronan fragments, and an aggrecan fragment among others [26–32]. Binding of DAMPs to TLR2 or TLR4 activates signaling pathways that are myeloid differentiation primary response gene 88 (MyD88)-dependent or MyD88-independent [33]. The downstream effects include activation of MAPK, PI3K and NFκB [33, 34] with the expression of proinflammatory cytokines and mediators e.g., inducible nitric oxide synthase (iNOS) and cyclooxygenase 2 (COX-2) [35, 36]. A role for innate immune response and TLR activation is suggested in the pathogenesis of OA [33, 37]. Chondrocytes derived from a TLR2/TLR4 double knockout mouse resisted the pro-catabolic effect of low molecular weight hyaluronan fragments and showed attenuated matrix metalloproteinase-13 (MMP-13) expression [38]. In RA, blocking TLR2 inhibits the spontaneous release of cytokines from synovial explant cultures [39]. Furthermore, TLR2 and TLR4 can act synergistically to upregulate inflammatory cytokine production from human RA synovial fibroblasts [40, 41].

Given the importance of TLR2 and TLR4 activation and signaling in OA and RA pathogenesis, we aimed to study whether PRG4 can interact with TLR2 and TLR4 and the functional consequence of this interaction. In this work, we present data supporting that PRG4, in its native and recombinant forms, can bind to immobilized TLR-2 and TLR-4. PRG4 exhibited concentration-dependent binding to TLR2 and TLR4. Furthermore, PRG4 might have exhibited a greater affinity towards binding TLR2 compared to TLR4, especially at the higher PRG4 concentrations evaluated. Using fluorescently labeled PRG, we have demonstrated that PRG4 can associate with cells that overexpress TLR2 and TLR4. In the cell association studies, PRG4 displayed better association with TLR2 expressing cells at the earlier time point examined. The binding of PRG4 to TLR2 and TLR4 may explain the concentration-dependent antagonism of TLR2 and TLR4 activation by bacterial cell wall ligands of the latter. The upstream effects of PRG4 in the TLR2 and TLR4 signaling cascade, namely blocking access to the TLR receptor, translated to inhibition of NFκB nuclear translocation. PRG4 alone did not alter the background level activation of TLR2 and TLR4 with no detectable receptor activation following incubation of the cells with PRG4 above that of background. Towards this end, PRG4 acts as a pure antagonist on the TLR2 and TLR4.

We observed that RA SF activated TLR2 and TLR4 to a greater extent compared to SF from subjects without history of joint arthropathy. We also report that the RA SF that we examined activates TLR2 to a greater extent compared to OA SF. These findings are in an agreement with previous studies in which RA SF activates TLR2-HEK and TLR4-HEK cells [42]. In our study, the magnitude of activation by RA SF was considerably lower than previously reported [42], which could be attributed to the significantly lower SF dilution used in our study. We also observed that OA SF activated TLR2 and TLR4. Previous reports have demonstrated that SF from patients with early OA augments the TLR ligand-mediated stimulation of FLS [43] and that plasma proteins present in OA SF stimulate cytokine production in a TLR4-dependent manner [44]. In our study, PRG4 was efficacious in blocking RA SF and OA SF induced TLR2 and TLR4 activation, with a lower PRG4 concentration required to block TLR2 compared to TLR4. Removing PRG4 from SF dramatically increased TLR2 activation by OA SF and RA SF. Additionally, PRG4 at a previously efficacious concentration that blocked TLR2-induced activation by OA SF and RA SF, had significantly reduced efficacy when used in OA SF and RA SF where PRG4 had been depleted. A higher PRG4 concentration was needed to block the excessive TLR2 stimulation of PRG4-immunoprecipitated OA SF and RA SF. By contrast, PRG4 removal from OA SF and RA SF had no effect on TLR4 activation, as PRG4-depeleted and control OA and RA SF demonstrated a similar pattern of TLR4 activation. This observed specificity of SF PRG4 towards TLR2 vs. TLR4 antagonism might be explained by the presence of higher fluid concentrations of TLR2 ligands than TLR4 ligands in the specimens we have studied. Additionally, preferential binding of PRG4 to TLR2 compared to TLR4 may also contribute to this observed specificity.

PRG4’s protein core is 1,404 amino acids long with N and C terminals and a central mucin domain [9]. The central mucin domain is heavily glycosylated via O-linked β(1-3)Gal-GalNAc oligosaccharides, and is configured to form a nanofilm that exerts repulsive forces, and provides the basis for its anti-adhesive and lubricating properties [45]. Removal of central domain glycosylation results in a loss of the boundary lubricating ability of PRG4 [46]. The nature of PRG4 glycosylation pattern may influence a biological role for PRG4 as altered glycosylation in PRG4 isolated from RA SF forms a ligand for L-selectin [19, 20, 47]. However, PRG4 binding to CD44 is not dependent on PRG4 glycosylation [18]. PRG4 amino terminal domains are homologous to the somatomedin B domain of vitronectin and the carboxy terminal contains a hemopexin domain [9] and may mediate surface binding of the protein [48]. The N and C terminals may allow PRG4 to simultaneously interact with multiple receptor families and modulate the downstream signaling of its binding partners. Unique to PRG4 is its ability to block NFκB activation downstream from two distinctly different receptor families, namely CD44 and the TLRs.

## Conclusions

In summary, we present data suggesting that PRG4 binds to TLR2 and TLR4 and associates with cells that overexpress these two receptors. In this interaction, PRG4 acts as an antagonist to prevent TLR2 and TLR4 activation by bacterial ligands and by a ligand present in SF from patients with inflammatory arthritis. An endogenous anti-inflammatory role for PRG4 is further characterized by increased TLR2 activation by OA and RA SF upon PRG4 removal and reversal of this effect following re-introduction of PRG4. This work, along with recent published reports [18, 20], identified a biological role for PRG4 that may be important to the onset and progression of chronic inflammatory joint diseases and provides a rationale for PRG4 as an anti-inflammatory agent.

## Abbreviations

ANOVA: analysis of variance
DAMP: damage-associated molecular pattern
FLS: fibroblast-like synoviocytes
HEK: human embryonic kidney
kDa: kiloDaltons
LPS: lipopolysaccharide
nhPRG4: native human proteoglycan-4
OA: osteoarthritis
PBS: phosphate-buffered saline
PRG4: proteoglycan-4
RA: rheumatoid arthritis
rhPRG4: recombinant human proteoglycan-4
SEAP: secreted embryonic alkaline phosphatase
SF: synovial fluid
TLR2: toll-like receptor 2
TLR4: toll-like receptor 4
